# Effect of Ultrasound and Enzymatic Mash Treatment on Bioactive Compounds and Antioxidant Capacity of Black, Red and White Currant Juices

**DOI:** 10.3390/molecules27010318

**Published:** 2022-01-05

**Authors:** Marcin Kidoń, Guruprasath Narasimhan

**Affiliations:** Department of Food Technology of Plant Origin, Poznań University of Life Sciences, 60-624 Poznań, Poland; prasath.guru@rocketmail.com

**Keywords:** currant berry, sonication, juice, anthocyanin, phenolics, ascorbic acid, antioxidant capacity

## Abstract

Ultrasound treatment is recognized as a potential technique for improvement in the nutritional values of fruit juices. This study was initiated with the objective of evaluating bioactive compounds and some important quality parameters of black (BC), red (RC) and white (WC) currant juices obtained from fruit mash preliminarily treated by enzymes combined with ultrasound. Individual and total phenolic content (TPC), anthocyanins, color parameters, ascorbic acid, antioxidant capacity (TEAC), juice yield, pH, titratable acidity, and soluble solids were investigated. Significant increases in the levels of TPC and antioxidant capacity of sonicated samples were observed. However, ultrasound treatment had no effect on individual phenolic compounds of juices. Sonication of mash before juice pressing did not cause any noticeable changes in ascorbic acid content. Only in the case of WC was an increase in content of vitamin C noticed. The color of juices obtained after treatment was similar to the control sample. It was demonstrated that enzymatic combined with ultrasound treatment of mash for different colored currant fruit did not have any dismissive effect and could even improve some parameters of the juice obtained.

## 1. Introduction

Currant fruit belongs to the genus *Ribes* cultivated in the temperate regions of Europe and North America, part of South America, Asia and Northwest Africa [1]. Currants are woody, perineal shrubs with almost 150 varying species. Currant berries are known for their attractive colors like black, red, and white (yellow) with unique flavors of sweet and tart. Berries are important sources of dietary fibers, micronutrients and phytochemicals [2]. The total global production of currants was around 650,000 tons for the last 10 years [3]. Currant fruits are consumed as eagerly as fresh ones, but are also found in the process of making juices, nectars, jams, jellies or syrups [4]. Raw fruits are very much susceptible to spoilage and might quickly undergo microbial deterioration. Therefore, juice production could be a preferable method, as well as a quick and an easy way to preserve the nutritional values of currant for a prolonged time.

One of the most common, predominant and commercially used species in fruit production is black currant (*Ribes nigrum* L.) [5]. This currant fruit has a color ranging from dark purple to black with a glossy skin and contains a calyx persisting at the apex part of the fruit. Black currant fruits are rich in bioactive substances, mainly phenolics and vitamin C. The amount of vitamin C in black currant ranges from 40 to even 310 mg/100 g of fresh fruit. Anthocyanins are the pigments responsible for the dark color of black currant fruit. There are four main anthocyanins found: delphinidin-3-*O*-glucoside, delphinidin-3-*O*-rutinoside, cyanidin-3-*O*-glucoside and cyanidin-3-*O*-rutinoside. Their content could reach up to 250 mg/100 g of fresh fruit which constitutes nearly 80% of the flavonoids in black currant [2,6,7,8].

The next most appreciated species is red currant (*Ribes rubrum* L.). Red currant berries are mainly consumed in an untreated form as a refreshing sour summer fruit. Also, they can be used for cooked dishes like tarts and for processing into juice, jam and jelly. The red color of the fruit is also caused by anthocyanins, but its content is lower compared to that of black currant and does not exceed 60 mg/100 g of fresh weight. The main anthocyanins identified in red currant are cyanidin-3-*O*-rutinoside, cyanidin-3-*O*-xylosylrutinoside and cyanidin-3-*O*-glucoside [9,10]. Also, vitamin C content is relatively low compared to that of black currant, whereas the content in the range from 20 to 70 mg/100 g is still appreciable from the nutritional point of view [2,7,9,11].

White currant (*Ribes sativum*) is the least popular species of currant. This fruit does not contain any anthocyanins, but is still considered as a valuable crop. White currant berries contain a high amount of hydroxybenzoic acid derivatives and proanthocyanidins [12]. White currant fruit can be considered as a source of ascorbic acid. The content of this compound could vary from 21 to 39 mg/100 g of fresh weight [13,14].

The advancement in science and technology over the years have enabled its development in the utilization of an innovative and versatile technology like ultrasound for the purpose of food processing, process control and preservation [15]. Some examples of applications are non-destructive examination of food composition, texture and other physicochemical features. During processing, ultrasound could generate emulsions or inactivate microbials in products or sterilize surfaces [16]. A few other studies have suggested that ultrasound is very useful for enhanced extraction of valuable compounds from plant materials [17,18]. A potentially promising application of ultrasound is in the field of juice processing, as it could improve nutrients content and facilitate preservation [19]. Sonication treatment of juice could enhance the amount of phenolic compounds in the case of some fruits, like pitch, grapefruit or strawberry [20,21,22].

However, the influence of ultrasound applied on mash before juice pressing on obtained juice parameters and bioactive compounds contents has not been well investigated and results obtained are not consistent. Some authors suggest that treating fruit tissue before juice extraction by ultrasound could disrupt the cell wall and might lead to increased juice yield, and could improve extraction of water-soluble compounds [21]. Positive results of such treatment in terms of yield, physicochemical features and antioxidant compounds in the case of mulberry were observed [23]. On the other hand, Radziejewska-Kubzdela et al. [24] in the case of barberry juice did not observe an increase of yield and ascorbic acid content in the juice obtained. Furthermore, the antioxidant capacity of the juices made from the mash subjected to sonication decreased by about 55% compared to raw material. To the best of our knowledge the studies about the influence of enzymatic combined with ultrasound treatment of the mash before juice production, especially in the case of different colored currant fruits, are limited. Also, enzymatic maceration combined with sonication has not been well examined yet. Therefore, the aim of this study was to evaluate the effect of ultrasound treatment of black, red and white currant fruits mash prior to juice pressing on bioactive compounds content, antioxidant capacity, yield and some quality parameters of the obtained juice.

## 2. Results and Discussion

### 2.1. Effect of Ultrasound Mash Treatment on Juice Yield

The minimization of use of raw material and reduction of waste is a very important aspect of juice production. It is considered that enzymatic and ultrasound treatment could increase the amount of juice yield. Juice yield of BC, RC and WC obtained from mash after enzymatic combined with ultrasound treatment is presented in Table 1.

Juice yield varied among currant varieties. The average juice yield was the highest in the case of RC (reached about 87%) among the three variety of currants that were treated. Followed by this, WC juice yield with 81% was lesser than the RC but reasonably more than that of BC. The lowest yield of juice was in BC with the average of only 72%.

Ultrasound combined with enzymatic mash treatment before juice pressing had an impact on the yield, but it was only a slight effect. In BC, the maximum amount of juice was obtained after 10 and 15 min of ultrasound mash treatment and was about 2.6 and 1.9 percentage points more than in the case of a sample without ultrasound mash treatment. The strongest effect was noticed in BC. For RC, the maximum amount of juice yield reached 88.0% by subjecting the mash to ultrasound for 15 min. In this case, the yield was 2.1 percentage points more compared to the control sample. A slight increase of juice yield with increased period of ultrasound treatment was observed in the case of WC. The maximum juice recovered from mash was observed after the ultrasound treatment for 15 min (treatment 3) and this yield was 1.3 percentage points more than that of yield from untreated sample. The reason for the low yield of juice in BC could be attributed to the high pectin content of berries, up to 2.5% [25]. During juice processing, pectin has a tendency to coagulation. This increased viscosity of juice and clogged intercellular species could decrease juice yield and pressing efficiency. Apart from ultrasound, in the all samples enzymatic mash treatment with pectinolytic enzyme was used. This was a necessary step because of high pectin content present in currants. Pectin is a substance that is responsible for mash cluster formation that leads the contents to clot and hence the amount of yield would initially be low or even impossible to press because of gelatinization [26,27]

Some other studies suggested that ultrasonic waves cavitation effect causes the disruption of fruit cell walls, surface erosion and some particle break down. That could lead to increased mass transfer and juice yield [28,29]. Also, the combination of enzymatic mash treatment along with ultrasound could further increase juice yield compared to enzymatic treatment alone [30].

### 2.2. Effect of Ultrasound Mash Treatment on pH, Titratable Acidity and Total Soluble Solids of Juice

The pH, titratable acidity and total soluble solids are the major quality and authenticity parameters of fruit juices which are responsible for their sensory characteristics. The results showed that ultrasound treatment of mash before juice pressing caused only slight changes in pH, titratable acidity and soluble solids (Table 2). The pH values of obtained juices ranged from 2.83 to 3.03. But in RC and WC, pH values did not show any changes, whereas in BC, pH increased from 2.83 to 3.03. Also, the titratable acidity of BC juices after 10 and 15 min of ultrasound mash treatment were higher than that of the untreated sample, or 5 min treated sample. The results showed that the highest differences among currant varieties was in case of titratable acidity. RC and WC juice samples possessed about 35% lower titratable acidity values then BC juice. By contrast with our results, Nour et al. [2] found that the titratable acidity of black and red currant varieties was similar in range. In connection with this, even among black varieties were the fluctuations by about 40%. Rubinskiene et al. [31] stated that titratable acidity decreased rapidly during ripening and could also be affected by weather conditions. The total content of soluble solid was found to be higher in WC and BC when compared to RC. The values of the samples showed some fluctuations in TSS, but there is no observed trend. Some authors suggest increasing TSS in juice after ultrasound treatment, because of an increase in the extraction efficacy due to the ultrasound treatment, causing the destruction of cell walls [30]. However, in our study TSS contents of the currant juices remained stable after treatment.

### 2.3. Total Phenolic Content (TPC) and Antioxidant Capacity (TEAC) of Juices

Currant fruit are valuable sources of phenolic compounds and ascorbic acid. BC in particular contained many phenolics, anthocyanins and ascorbic acid. These compounds have the potential to scavenge the free radicals and thereby could reduce oxidative stress. The amount of TPC in BC ranged from 447 mg/100 mL to 538 mg/100 mL. Ultrasound mash treatment had a positive influence on the TPC of the juice. Nonetheless, 5 min treatment caused an 8% increase in the TPC compared to the untreated sample. On extending the treatment time further by 5 min the TPC increased by another 8% and reached a value of 534 mg/100 mL. On further increasing the sonication time, only a minor effect on TPC was observed (Table 3).

RC also contained a considerable amount of phenolic compounds which was strongly influenced by ultrasound. After the first 5 min of ultrasound treatment, there were no major changes in TPC (Table 3). It might be that in RC the treatment of 5 min was not enough to release phenolic compounds. However, after 10 min of ultrasound, there was a significant increase by 31% in TPC which further increased by 58% by 15 min of ultrasound treatment.

A different trend was observed in WC juice. The highest value of TPC was found in the sample without ultrasound mash treatment. After ultrasound mash treatment for 5 min there was a decline in the TPC of the juice by nearly 14%. However, further extending the time from 5 min to 10 min, there was a 4% increase observed and after extension from 5 to 15 min this was 7%. Even the WC juice possessed higher values of TPC compared with other currant cultivars.

Ultrasound is a promising technique for improving the recovery of phenolic compounds during extraction [17,32]. As noticed by others, ultrasound assistance for a duration of 15 min in black chokeberry enhanced the yield of phenolic extracts up to 85% [18]. Also, it was responsible for the production of antioxidant-rich extracts with reduced time and energy. The increase of TPC in BC and RC juices after ultrasound treatment of mash could be explained by acoustic cavitation, which causes swelling of cells during sonication. This leads to an increase in the diffusion rates of cell contents into the external juice [33]. Another possible explanation for observed changes might be due to the chemical changes of phenolic compounds. Sonication could generate hydroxyl radicals (OH-) which could attach to the aromatic ring and generate a different chemical structure of phenolics. Responses of assay with Folin–Ciocalteau reagent to different compounds are not specific they and could decrease or increase [22,34].

Antioxidant capacity of obtained juices varied from 20.1 to 42.4 micromol Trolox per mL of juice (Table 3). The antioxidant capacity was about two-fold higher for WC and BC juices compared to that of RC juice. However, after ultrasound mash treatment a significant increase in antioxidant capacity in all currant varieties was noticed. In almost all cases, after 10 min of sonication increase of antioxidant capacity reached at least 10%. The RC sample observed an increase after 10 and 15 min that was about 20%. In WC, juice obtained after 5 and 10 min of mash treatment showed about 13% more antioxidant capacity than the control sample. It was also found that the antioxidant capacity of the juices was positively correlated with TPC (R^2^ = 0.69).

Ultrasound treatment of mash before juice pressing or juice sonication after pressing could improve the antioxidant properties of final products. Nguyen and Nguyen [23] observed that the ultrasonic treatment before juice pressing increase antioxidant capacity by about 41% in the case of the mulberry. However, some authors noticed that longer sonication time or too extensive ultrasound power could have the opposite effect and could diminish antioxidant capacity and degrade bioactive compounds. That could be explained by an increasing generation of hydroxyl radicals. These radicals could reduce the content of bioactive compound and antioxidant activity [35,36]. While Radziejewska-Kubzdela et al. [24] noticed that antioxidant capacity of barberry juices made from untreated mash and from mash subjected to sonication was not statistically different.

### 2.4. Determination of Phenolic Compounds by High-Performance Liquid Chromatography (HPLC)

Currant fruits are well known sources of various phenolic compounds. Different subgroups of phenolic compounds are identified by others. The main ones are anthocyanins, flavonols, flavan-3-ols and hydroxycinnamic acid derivates [37]. A summary of all the identified phenolic compounds for studied currant juices is given in Table 4. Chromatographic retention, spectral characteristics, external standards and literature data were used to support the identification of the peaks. Chlorogenic, neochlorogenic and *p*-coumaric acids were analyzed to be the major phenolic compounds in BC juice based on available standards. Also, gallic acid and quercetin derivatives were identified in considerable amounts. The main phenolic acid for RC and WC juices was gallic acid. Apart from that, hydroxycinnamic acids were also identified in high amounts. Chlorogenic and neochlorogenic acids were from 18% to 22% of all phenolic compounds found in RC and WC juices. Results regarding the effect of sonication treatments presented in Table 4 revealed only slight changes in chromatograms and phenolic content. Only in BC juice after sonication of mash did the sum of phenolic compounds increase by about 6–7%. A small increase of individual phenolic compounds after ultrasound mash treatment confirms the research of Olawuyi et al. [38] in plum juice. Pingret et al. [17] noticed that ultrasounds in the extraction of apple pomace could increase the extraction of dihydrochalcones and phenolic acids. On the other hand, the bonds between phenolics and polysaccharides could not be broken, thus some polymeric compounds could not be released.

### 2.5. Determination of Anthocyanin by HPLC

Anthocyanins are relevant to juice quality in terms of color as well as antioxidant properties. All BC juice samples possessed higher anthocyanins content than RC juices. Because of the light color of WC juice, anthocyanins were not examined. Total anthocyanins content were in ranges from 135 to 147 mg/100 mL and from 17.0 to 17.9 mg/100 mL for BC and RC juices, respectively (Table 5). Ultrasound treatment had a negligible effect on anthocyanin content. Tiwari et al. [39] did not observe any increase of anthocyanin content in grape juice after ultrasound treatment. Furthermore, prolonged treatment at higher power levels may induce a decrease of anthocyanins probably due to its chemical decomposition. Alighourchi et al. [40] did not observe any trend in the anthocyanins content in pomegranate juice after sonication. The differences in anthocyanin content ranged from −8.41 to +6.84% depending on the amplitudes and time of ultrasound treatment. Olawuyi et al. [38] found a higher content of anthocyanins in plum juice after enzymatic treatment or enzymatic combined with ultrasound-treated samples. Samples treated only with ultrasound had lower values of anthocyanin content, similar to control sample. The authors explained that by the inhibition of polyphenol oxidase enzyme activity by enzymatic and ultrasound treatment which could reduce the hydrolysis of anthocyanins.

In agreement with other studies on BC juices, the four main anthocyanins (delphinidin and cyanidin derivatives) were identified (Table 5.). Other authors found trace amounts of petunidin, peonidine and malvinidine derivatives [7,10,41]. HPLC analysis revealed six peaks in RC juice. According to the retention time and spectra characteristics delphinin-3-*O*-glucoside, cyanidin-3-*O*-glucoside and cyanidin-3-*O*-rutinoside were confirmed (Table 5). Some other studies suggested the presence of cyanidin di- and triglycosides, such as cyanidin-3-*O*-sophoroside, cyanidin-3-*O*-rutinoside, cyanidin-sambubiosyl-rhamnoside or cyanidin-3-*O*-(2-xylosyl)rutinoside [7,10].

### 2.6. Instrumental Color Parameters of Currant Juices

Color is an important factor contributing to the quality attribute of fresh and processed food products and juices and is also responsible for consumer choice, perception and decisions in buying the product. Instrumental color parameters of BC, RC and WC juices obtained after different durations of ultrasound mash treatment are presented in Table 6.

Instrumental color analysis of all samples showed that the L* values of WC were found to be the highest and a* values were the lowest among all three currant fruit juices. Moreover, as the values of a* and b* in all treatments in all the three currants were positive, this means that the hues of the color were red and yellow respectively. a* was observed to be at the maximum in RC, followed by BC and had very low values in WC. b* was found to be at the maximum in BC, then RC and the lowest in case of WC. WC does not contain anthocyanin pigments that could explain light color of obtained juice.

From the results obtained, the impact of ultrasound treatment of mash before juice pressing was negligible. In BC juices, the highest color differences between control and other samples were found (ΔE varied from 1.4 to 4.9). For other samples color changes were not higher than 2, which could generally be considered to be barely perceptible by the average human observer. Even differences from 3 to 5 can be noticed by the untrained human eye, which is very small and acceptable for commercial products [42].

Also, Aadil et al. [20] checked the color parameters of grape fruit juices exposed to the sonication. The color difference estimated between the longest treated (90 min) and control sample juices was just 1.2. The pressure developed during the sonication treatment could enhance homogenization. Thus, sonication treatment could improve the consistency and uniformity of liquid products [20]. It is suggested that the sonication might be used for the processing of juices without noticeable changes in color.

### 2.7. Ascorbic Acid Content in Juices

Ascorbic acid (or vitamin C) is a water soluble vitamin. The human body is not able to produce this vitamin, hence it is essential to provide an appropriate amount of ascorbic acid with our diet. Currant berries, especially BC, are very rich sources of vitamin C. Average contents of ascorbic acid in juice samples obtained from ultrasound treatment of mash were about 107, 15 and 5 mg/100 mL for BC, RC and WC, respectively. Huge differences in ascorbic acid content among the different colored currant cultivars were also found by other authors. Some values are even two- or three-fold higher than in our study [2,10,14]. Ascorbic acid content varied among cultivars, but also is influenced by other factors like growing, weather or storage conditions as well as pH, temperature or light exposure. This could explain the differences between own results and amounts reported by others.

Sonication of mash before juice pressing in case of BC and RC varieties did not cause noticeable changes in ascorbic acid content (Table 3). While in the case of WC increase in the content of vitamin C by 58% after 5 min of ultrasound mash treatment was noticed. The influence of ultrasonic waves on ascorbic acid could be indecisive. Some authors suggested the increase in vitamin C could be ascribed to the removal of entrapped oxygen due to cavitation [43]. A previous study conducted on sonicated grapefruit juice showed some increase in vitamin C content of the juice after 30, 60 and 90 min of treatment [20]. Nguyen and Nguyen [23], after applying ultrasonic treatment for 60 min, observed significantly increased ascorbic acid content in mulberry juice by about 50% compared to the pressed juice only. However, when the ultrasonic time was prolonged, ascorbic acid content started to reduce. During ultrasound processing, free radicals, hydrogen ions and hydrogen peroxide could be formed. These molecules could cause oxidation reactions and promote degradation of ascorbic acid during sonication. Adekunte et al. [44] found that the sonication of tomato juice for 10 min diminished vitamin C content by about 32%.

## 3. Materials and Methods

### 3.1. Currant Fruit

The three kinds of currant fruits: black currant [BC] (cv. Öjebyn), red currant [RC] (cv. Jonkheer van Tets) and white currant [WC] (cv. Weißea us Jüterbog in English: White from Juterbog) were used. Plants were cultivated on a private farm in central Poland (Ziemia Łódzka district, Sieradz county) in 2018. Ripe currant fruits were manually harvested in June, destemmed, packed in plastic bags in portions of 500 g each, and frozen at −18 °C. Fruit were stored in a freezer before being used for juice preparation.

### 3.2. Enzymatic and Ultrasound Mash Treatment

**Figure 1:**
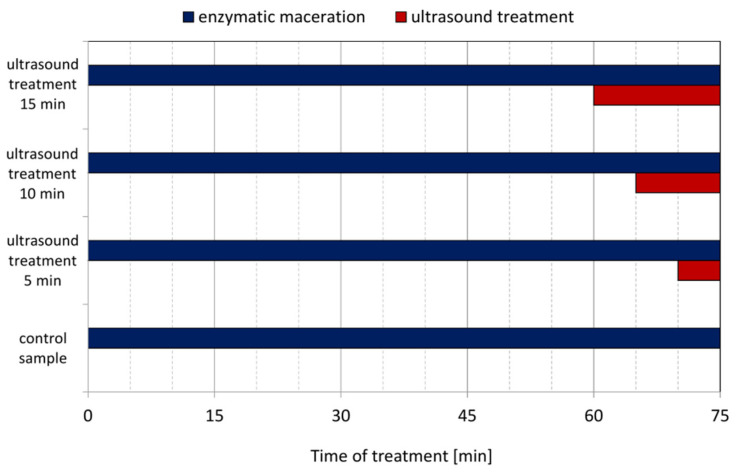
Times of enzymatic and ultrasound treatments of currant mash before juice pressing.

Enzymatic combined with ultrasound treatment of mash was conducted in the ultrasound water bath SW3H (Sonoswiss AG, Ramsen, Switzerland). This equipment operated at a frequency of 37 kHz with ultrasonic effective power of 80 W. The currant fruits were defrost at room temperature. Completely thawed fruits were crushed in a Thermomix TM 3 (Vorwerk SE and Co. KG, Wuppertal, Germany) mixer at 50 °C for 8 min at speed level 5. After crushing, portions of the mashed fruits were weighed at about 500 g (exact mass of portion was noted), transferred and evenly distributed into the stainless-steel chamber of the ultrasound water bath. Enzyme Fructozym EC color (Erbslöh Geisenheim GmbH, Geisenheim, Germany) was used for enzymatic mash treatment. Mash maceration is an important step in currant juice production. It cause improved press and avoid gelatinization of the juice. Enzymatic maceration conditions, dosage and time were set according to enzyme producer guidelines. To the crushed currant, the enzyme was added (0.3 mL/kg of mash), and this was mixed and then placed in a water bath at 50 °C without ultrasound in order to allow the enzyme to work. After a specific time, the ultrasound bath was set to maximum ultrasound power and mashes were sonicated for the durations shown in Figure 1. Total time of processing of each samples was 75 min.

### 3.3. Juice Pressing

Mash after treatment was transferred to the cage of the hydraulic press Para-Press (Paul Arauner GmbH, Kitzingen, Germany). The pressing pressure was produced by tap water (3.0 ± 0.1 bar) by means of a rubber bladder for 5 min. Juice was collected in a plastic bucket and weighed. Then, the juice was transferred to a plastic bottle and frozen at −50°C then stored in that condition until analysis.

### 3.4. Evaluation of Juice Yield

Mashes before the juice production and obtained juice were weighed on a laboratory scale. The juice yields were calculated from the formula as follows:

(1) $$\mathrm{juice}\;\mathrm{yield}\;\left[\%\right]=\frac{\mathrm{mass}\;\mathrm{of}\;\mathrm{the}\;\mathrm{juice}\;\left[g\right]}{\mathrm{mass}\;\mathrm{of}\;\mathrm{the}\;\mathrm{mash}\;\left[g\right]}\times 100\%$$

### 3.5. Determination of pH, Titratable Acidity and Soluble Solids

Analysis of pH was conducted with pH-meter Orion model 710A (Thermo Fisher Scientific, Waltham, MA, USA). Titratable acidity (TA) determination of the juice samples was done using titration method with 0.1 M NaOH. The total acidity was expressed in g citric acid/L. The total soluble solids (TSS) were determined in an optical refractometer, model HI96801 (Hanna Instruments, Smithfield, RI, USA) and expressed as percentage of sucrose (°Bx).

### 3.6. Total Phenolic Content (TPC)

The total phenolic content was assessed using the Folin–Ciocalteu method [45]. Prior to the analysis, the juice samples were centrifuged at 5000× *g* for 10 min. The results were expressed as mg gallic acid equivalents/100 mL (GAE).

### 3.7. Determination of Phenolic Compounds by HPLC

Determination of phenolic compounds was carried out on an Agilent Technologies 1200 Rapid Resolution system (Agilent, Santa Clara, CA, USA) equipped with degasser, binary pump, autosampler holder, column holder and diode array detector (DAD). For chromatographic resolution, Zorbax SB C-18, 5 µm, 4.6 × 150 nm column was used. The mobile phases were (A) 6% acetic acid in 2 mM sodium acetate (*v*/*v*) and (B) acetonitrile. The elution gradient was linear as follows: 0–15 min: 0–5% B; 15–25 min: 5–20% B; 25–30 min: 20–30% B; 30–35 min: 30–50% B; 35–40 min: 100% B. Juice samples were centrifuged at 5000× *g* for 10 min, appropriately diluted with distilled water, and filtered through 0.45 µm PTFE syringe filters. Gallic acid was quantified at 280 nm and other phenolics at 320 nm. Gallic acid and chlorogenic acid were used as external standards.

### 3.8. Determination of Anthocyanins by HPLC

The anthocyanin content of juices was determined using HPLC (Agilent, Santa Clara, CA, USA) as described by Oszmiański and Sapis [46]. Juices before analysis were centrifuged at 5000× *g* for 10 min and diluted with distilled water. The same HPLC system as for phenolic compounds determination was used. The mobile phases were: (A) 10% formic acid in water (*v*/*v*) and (B) formic acid/acetonitrile/water (1/3/6; *v*/*v*/*v*). The elution gradient was linear as follows: from 0 to 15 min, the solvent B increased from 20 to 50%, from 16 to 20 min, it increased up 100%, from 20 to 21 it remained at 100% and from 21 to 23 min it decreased to 20%. The detector was set for scanning in the range of 400 to 700 nm. Quantification was undertaken at 520 nm and calculated as cyanidin-3-*O*-glucoside.

### 3.9. Color Measurement

The color parameters of the currant fruit juice samples were measured using a spectrophotometer Konica-Minolta CM-3600 d (Konica Minolta Inc., Tokyo, Japan). The color parameters were expressed in terms of the CIE L*a*b* system. Measurement parameters were as follows: measurement in transmittance, the observer angle 10°, illuminant D65 as light source. Measurements were conducted in a glass cuvette with optical length of 10 mm.

### 3.10. Determination of Ascorbic Acid Content by HPLC

Ascorbic acid content determination was performed according to [47]. Currant juices were mixed with 1% meta-phosphoric acid. To reduce dehydroascorbic acid into ascorbic acid form before chromatographic separation, dithiothreitol was used. The HPLC system was the same as described in Section 3.7. The mobile phase was 1 mM potassium dihydrogen phosphate in water and methanol. The detector was set for scanning in the range of 200 to 380 nm. Quantification was undertaken at 245 nm.

### 3.11. Antioxidant Capacity Determination (TEAC)

The antioxidant capacity was determined using ABTS^●+^ assay following the procedure of Re et al. [48]. The results were expressed as micromoles of Trolox equivalent antioxidant capacity per mL of juice (TEAC).

### 3.12. Statistical Analysis

The analysis of variance (ANOVA) was used to determine the significance of the main effects. Tukey’s post hoc test was used to determine differences between the mean values of multiple groups. Correlations were analyzed with Pearson’s test. Statistical significance was set at *p* < 0.05. The Statistica 13.1 software (TIBCO Software Inc., Palo Alto, CA, USA) and Excel 2010 (Microsoft Corporation, Redmond, WA, USA) was used for the analyses.

## 4. Conclusions

Treating the mash of different colored currants by ultrasound combined with an enzyme before juice pressing showed improvement in TPC and antioxidant capacity. However, a slight increase of juice yield was observed. Small differences in other parameters such as pH, acidity, soluble solids, color, ascorbic acid content and individual phenolic compounds of juices obtained from mash subject to ultrasound combined with enzyme treatment before pressing were also observed. This was except for the case of BC sample, where the sum of phenolic compounds content increased by about 6–7%, and the WC sample, where vitamin C content increased by 58%. Enzymatic combined with ultrasound treatment did not have dismissive effects on bioactive compounds and other features of the juices obtained.

## Figures and Tables

**Table 1:** Yield of black currant (BC), red currant (RC) and white currant (WC) juice obtained after different durations of ultrasound mash treatment.

Duration of Ultrasound Treatment [min]	Juice Yield [%]		
	BC	RC	WC
0	71.2	85.9	80.6
5	68.9	86.5	80.7
10	73.8	86.3	81.4
15	73.1	88.0	81.9

**Table 2:** pH, titratable acidity and total soluble solids in BC, RC and WC juices obtained after different durations of ultrasound mash treatment (different letters in the same column indicate significant differences between data *p* < 0.05).

Duration of Ultrasound Treatment [min]	pH	Titratable Acidity [g/L]	Total Soluble Solids [°Bx]
BC			
0	2.83 ± 0.02a	42.1 ± 0.2a	12.7 ± 0.1b
5	2.88 ± 0.07ab	41.0 ± 0.5a	12.2 ± 0.1a
10	3.00 ± 0.03bc	48.0 ± 0.0c	12.1 ± 0.1a
15	3.03 ± 0.03c	45.0 ± 0.2b	12.6 ± 0.2b
RC			
0	2.98 ± 0.02a	27.8 ± 0.5a	11.0 ± 0.0b
5	2.96 ± 0.03a	32.0 ± 0.9b	10.8 ± 0.1a
10	2.95 ± 0.02a	30.1 ± 0.9ab	11.2 ± 0.1c
15	2.96 ± 0.03a	29.1 ± 0.0a	10.7 ± 0.1a
WC			
0	3.00 ± 0.02a	27.2 ± 0.5a	12.6 ± 0.1ab
5	3.02 ± 0.03a	26.6 ± 0.0a	12.4 ± 0.1a
10	3.01 ± 0.05a	27.0 ± 0.7a	12.4 ± 0.2ab
15	3.01 ± 0.02a	26.9 ± 0.5a	12.8 ± 0.2b

**Table 3:** Total phenolic (TPC), ascorbic acid content and antioxidant capacity of ABTS assay (TEAC) of BC, RC and WC juices obtained after different durations of ultrasound mash treatment (different letters in the same column indicate significant differences between data *p* < 0.05).

Duration of Ultrasound Treatment [min]	TPC [mg/100 mL]	Ascorbic Acid [mg/100 mL]	Antioxidant Capacity TEAC [mmol/mL]
BC			
0	447 ± 32a	104.6 ± 1.8a	35.8 ± 1.2a
5	499 ± 5ab	106.5 ± 1.4a	36.4 ± 1.1a
10	534 ± 6b	108.1 ± 1.8a	38.3 ± 2.5ab
15	538 ± 12b	108.2 ± 2.5a	40.3 ± 1.8b
RC			
0	310 ± 14a	15.4 ± 3.3a	20.1 ± 0.1a
5	305 ± 10a	13.2 ± 0.7a	20.7 ± 2.4ab
10	401 ± 29b	16.5 ± 3.8a	24.7 ± 1.8b
15	490 ± 50c	14.7 ± 3.1a	23.9 ± 0.6b
WC			
0	569 ± 9b	3.4 ± 0.5a	37.1 ± 1.7a
5	493 ± 31a	5.4 ± 0.4b	42.1 ± 1.8b
10	513 ± 29ab	5.1 ± 0.5b	42.4 ± 2.1b
15	550 ± 22b	6.1 ± 1.8b	41.0 ± 1.4ab

**Table 4:** Individual phenolics content [mg/100 mL] of BC, RC and WC juices obtained after different durations of ultrasound mash treatment (nd–not detected).

Duration of Ultrasound Treatment [min]	Gallic Acid	Neochlorogenic Acid	Chlorogenic Acid	Quercetin Derivative 1	Quercetin Derivative 2	Quercetin Derivative 3	*p*-Coumaric Acid	Sum of Unknown	Sum of Phenolics
BC									
0	2.02 ± 0.09	4.21 ± 0.14	4.18 ± 0.20	2.22 ± 0.08	2.02 ± 0.06	0.84 ± 0.05	3.56 ± 0.09	9.04 ± 0.64	30.1 ± 0.9
5	1.90 ± 0.13	4.28 ± 0.17	4.79 ± 0.17	2.21 ± 0.15	2.06 ± 0.16	0.93 ± 0.06	4.03 ± 0.13	9.98 ± 0.58	32.2 ± 1.1
10	1.54 ± 0.03	4.20 ± 0.23	4.84 ± 0.13	2.33 ± 0.14	2.08 ± 0.15	0.93 ± 0.02	4.01 ± 0.20	10.0 ± 0.9	32.0 ± 1.1
15	1.55 ± 0.10	4.27 ± 0.20	4.96 ± 0.19	2.40 ± 0.15	2.21 ± 0.05	1.03 ± 0.03	3.87 ± 0.25	9.52 ± 0.68	31.9 ± 1.4
RC									
0	2.81 ± 0.02	1.12 ± 0.06	0.91 ± 0.03	nd	0.68 ± 0.04	0.18 ± 0.06	0.29 ± 0.04	2.19 ± 0.18	9.26 ± 0.24
5	2.92 ± 0.07	1.18 ± 0.08	0.82 ± 0.07	nd	0.67 ± 0.05	0.10 ± 0.07	0.31 ± 0.04	2.34 ± 0.10	9.60 ± 0.65
10	2.96 ± 0.04	1.09 ± 0.04	0.84 ± 0.05	nd	0.70 ± 0.08	0.15 ± 0.06	0.33 ± 0.03	2.70 ± 0.20	9.94 ± 0.60
15	2.92 ± 0.02	1.14 ± 0.03	0.95 ± 0.07	nd	0.72 ± 0.05	0.18 ± 0.04	0.32 ± 0.03	2.37 ± 0.16	9.88 ± 0.65
WC									
0	4.72 ± 0.03	2.56 ± 0.08	0.89 ± 0.04	0.61 ± 0.03	0.25 ± 0.03	0.05 ± 0.04	0.40 ± 0.05	7.01 ± 0.58	18.3 ± 0.9
5	4.69 ± 0.06	2.55 ± 0.07	0.90 ± 0.04	0.61 ± 0.04	0.24 ± 0.06	0.04 ± 0.05	0.50 ± 0.07	7.41 ± 0.68	18.7 ± 0.9
10	4.67 ± 0.02	2.51 ± 0.08	0.89 ± 0.06	0.62 ± 0.02	0.18 ± 0.07	0.00 ± 0.06	0.41 ± 0.07	7.67 ± 0.56	18.8 ± 0.9
15	4.73 ± 0.05	2.54 ± 0.04	0.93 ± 0.08	0.60 ± 0.03	0.22 ± 0.06	0.05 ± 0.04	0.45 ± 0.02	6.33 ± 0.37	17.7 ± 0.8

**Table 5:** Anthocyanin pigments content [mg/100 mL] of BC and RC juices obtained after different durations of ultrasound mash treatment (nd–not detected; different letters in the same column indicate significant differences between data *p* < 0.05).

Duration of Ultrasound Treatment [min]	Delphinidin-3-*O*-Glucoside	Delphinidin-3-*O*-Rutinoside	Cyanidin-3-*O*-Glucoside	Cyanidin-3-*O*-Rutinoside	Sum of Unknown	Sum of Anthocyanins
BC						
0	20.9 ± 0.4	62.2 ± 0.7	8.1 ± 0.1	49.0 ± 0.5	nd	140 ± 1a
5	21.0 ± 0.2	63.2 ± 0.9	7.8 ± 0.1	49.2 ± 0.9	nd	141 ± 2a
10	22.2 ± 0.3	65.2 ± 0.4	8.6 ± 0.1	51.5 ± 0.2	nd	147 ± 1a
15	22.6 ± 1.3	59.4 ± 10.2	8.3 ± 0.5	44.6 ± 7.4	nd	135 ± 19a
RC						
0	0.18 ± 0.01	nd	6.2 ± 0.1	2.1 ± 0.1	8.7 ± 0.1	17.1 ± 0.1a
5	0.18 ± 0.01	nd	6.1 ± 0.1	2.1 ± 0.1	8.6 ± 0.1	17.0 ± 0.1a
10	0.19 ± 0.01	nd	6.4 ± 0.1	2.2 ± 0.1	9.0 ± 0.1	17.9 ± 0.1a
15	0.18 ± 0.01	nd	6.2 ± 0.2	2.1 ± 0.1	8.6 ± 0.2	17.1 ± 0.5a

**Table 6:** Instrumental color parameters of BC, RC and WC juices obtained after different durations of ultrasound mash treatment (different letters in the same column indicate significant differences between data *p* < 0.05).

Duration of Ultrasound Treatment [min]	L*	a*	b*	ΔE
BC				
0	22.8 ± 0.1d	55.0 ± 0.1d	39.2 ± 0.1d	-
5	22.2 ± 0.1c	54.3 ± 0.1c	38.2 ± 0.2c	1.4
10	20.6 ± 0.1a	52.6 ± 0.1a	35.5 ± 0.1a	4.9
15	21.2 ± 0.1b	53.6 ± 0.1b	36.5 ± 0.2b	3.5
RC				
0	62.1 ± 0.4ab	60.3 ± 0.3a	20.8 ± 0.3ab	-
5	62.5 ± 0.1b	60.0 ± 0.1a	20.4 ± 0.1a	0.7
10	61.6 ± 0.1ab	61.1 ± 0.1b	21.9 ± 0.1c	1.5
15	62.1 ± 0.1a	60.4 ± 0.1a	21.1 ± 0.1b	0.3
WC				
0	93.4 ± 0.1bc	0.9 ± 0.2a	7.8 ± 0.1a	-
5	93.6 ± 0.1c	0.6 ± 0.1a	7.8 ± 0.1a	0.3
10	93.4 ± 0.1b	0.6 ± 0.1a	8.0 ± 0.1b	0.4
15	93.1 ± 0.1a	0.8 ± 0.1a	8.7 ± 0.1c	1.0

## Data Availability

Data are available from the authors on request.

## Sample Availability

Samples of the compounds are not available from the authors.
